# How Much Reliable Is the Current Belief on Grade Group 1 Prostate Cancer?

**DOI:** 10.3389/pore.2021.629489

**Published:** 2021-04-13

**Authors:** Mun Su Chung, Yeong Jin Choi, Young Sub Lee, Byung Il Yoon, U-Syn Ha

**Affiliations:** ^1^ Department of Urology, International St. Mary’s Hospital, Catholic Kwandong University, Incheon, South Korea; ^2^ Department of Hospital Pathology, Seoul St. Mary’s Hospital, College of Medicine, The Catholic University of Korea, Seoul, South Korea; ^3^ Department of Hospital Pathology, Eunpyeong St. Mary’s Hospital, College of Medicine, The Catholic University of Korea, Seoul, South Korea; ^4^ Department of Urology, Seoul St. Mary’s Hospital, College of Medicine, The Catholic University of Korea, Seoul, South Korea

**Keywords:** prostatic neoplasms, prostatectomy, neoplasm grading, gleason score, neoplasm invasiveness

## Abstract

**Objective:** To evaluate the clinicopathological characteristics of grade group 1 (GG1) prostate cancer in Korean populations.

**Methods:** We retrospectively analyzed 492 consecutive radical prostatectomy specimens from our institution, which included those from 322 men with clinical GG1 and 170 with clinical GG2 tumors between years 2009 and 2018. The incidence of Gleason score (GS) upgrading, extraprostatic extension (EPE), and seminal vesicle invasion (SVI) were evaluated in patients with clinical GG1. In pathological GG1 cases, the distribution of adverse pathological features including EPE, lymphovascular invasion (LVI), perineural invasion (PNI), and biochemical recurrence (BCR) was analyzed.

**Results:** Altogether, 78 (24.2%) out of 322 men in the clinical GG1 group demonstrated upgrading of GS, including 19 men with pathological Gleason score 4 + 3 = 7 and 6 with ≥ pathological Gleason score 4 + 4 = 8 cases. EPE was found in 37 (11.5%) and 22 (8.9%) men in clinical GG1 and pathological GG1 group, respectively. The incidence of LVI and PNI in the pathological GG1 cases was 2.8% (n = 7) and 28.6% (n = 71), respectively. BCR was observed in 4 men in pathological GG1 T2 (n = 226) and 2 men in GG1 T3 (n = 22) group. When we compared the pathological features between pathological GG1 T3 vs. GG2 T2, there was no statistical differences in the incidence of LVI and PNI between the two groups.

**Conclusions:** Contrary to the current concept that GG1 is almost always clinically insignificant, it seems that GG1 still possess its respectable position as a group of cancer with aggressiveness. These findings should be kept in mind when deciding on treatment options for prostate cancer patients in the Asian populations.

## Introduction

Recently the Gleason system has been compressed into so-called grade groups (GGs). The new GG system was validated in two large cohorts (men treated with radical prostatectomy (RP) or radiation), and both studies discovered that GGs predicted the risk of recurrence following the primary treatment [1, 2]. In the larger study, the five-year biochemical recurrence (BCR)-free progression probabilities after RP for GGs 1 through 5 were 96% (95% confidence interval (CI), 95–96), 88% (95% CI, 85–89), 63% (95% CI, 61–65), 48% (95% CI, 44–52), and 26% (95% CI, 23–30), respectively [1]. Other studies also supported the validity of this new system [3, 4]. Therefore, many experts believe that the International Society of Urological Pathology (ISUP) GGs will enable patients to better understand their true risk level. In 2019, the National Comprehensive *Cancer* Network panels have accepted the new GG system to conduct better treatment discussions compared to those using Gleason score (GS).

From this background, GG1 disease is increasingly being believed to be an insignificant prostate cancer which does not require immediate treatment due to its long natural history and low metastatic potential [5–7]. It is highly possible that such concept on GG1 affects physicians’ decision-making on treatment options, although most AS protocols include a combination of several factors (such as prostate specific antigen (PSA), PSA density, number of positive cores, maximal core percentage, etc) and GG, not GG alone.

Furthermore, several authors have suggested to remove the label “cancer” from GG1 lesion. Instead, they proposed indolent lesion of epithelial origin as the potential alternative term for this tumor [8–10]. This was based on several reports demonstrating extremely rare incidence of extraprostatic extension (EPE) and the lack of both seminal vesicle invasion (SVI) and metastatic potential with GG1 cases [11].

Moreover, there is a lack of literature analyzing prostate cancer patients among Asian population. Therefore, we aimed to evaluate the characteristics of both clinical and pathological GG1 cases among the Korean population.

## Materials and Methods

### Demographics and Clinicopathological Data

A total of 1,590 consecutive Korean patients with localized or locally advanced prostate cancer who were treated by RP between 2009 and 2018 were selected from our institution. Among these individuals, patients with incomplete medical records were excluded, as well as patients who received preoperative androgen deprivation or radiation therapy.

We obtained data on patient demographics and clinical characteristics, including age, body mass index, prostate volume on transrectal ultrasound, preoperative PSA values, clinical T stage, biopsy and pathological GS, the presence of EPE, SVI, lymphovascular invasion (LVI) and perineural invasion (PNI), and biochemical recurrence (BCR).

Pathological outcomes were assessed under the American Joint Committee on *Cancer* (AJCC) 2010 staging system [12], and tumor grading was classified using the ISUP 2014 Gleason grading system [13]. *Clinical GG* was defined as GG determined from prostate biopsy (preoperative GG) and *pathological GG* was defined as GG determined from radical prostatectomy specimen (e.g. final pathology). All RP specimens were weighed, inked, fixed overnight in ambient formalin, cut at 3 mm intervals, and submitted as quadrants. All procedures were performed by an experienced genitourinary pathologist (Y.J.C). Since the determination of patients’ GSs in 2009–2015 had been based on the pre-ISUP 2014 classification system in our institution, we needed to verify all pathological slides in this period to determine which patient had or did not have any cribriform patterns in their biopsy and RP pathologies (Gleason score 3 + 3 = 6 tumors, which were determined in the pre-ISUP 2014 era). This evaluation was also performed by Y.J.C. GG1 tumor in this study included Gleason score 3 + 3 = 6 tumor only (not tumor ≤ Gleason sum 5). BCR was defined as two consecutive rises in PSA after the post-treatment nadir, with the last PSA 0.2 ng/ml or higher.

### Study Endpoints

The primary objective of the current study was to analyze the incidence of GS upgrading and upstaging at RP in patients with *clinical GG1* cancer. The secondary objective is to determine the feature of *GG1 itself* (*pathological GG1*). Here, we analyzed the distribution of adverse pathological features, including locally advancing, LVI, PNI, and BCR in *pathological GG1* cases.

### Statistical Analyses

The patients’ preoperative and pathological characteristics were calculated using means for continuous variables and proportions for categorical variables. Student’s t-test and Mann-Whitney-Wilcoxon test were used for continuous variables, and the chi-square test and Fisher’s exact test were used to compare categorical variables between groups. To calculate BCR-free survival, Kaplan-Meier survival analysis with the log-rank test was used. Statistical analyses were performed with R statistics version 3.5.1. Values of *p* < 0.05 were considered to indicate statistical significance.

### Ethics Statement

The present study was approved by the Institutional Ethics Committee of Catholic Kwandong University College of Medicine after reviewing the study protocol and procedures (IS20RIMI0009). The requirement for written consent was waived because of the retrospective nature of the study. The data were anonymized prior to the analysis.

## Results

### General Clinical Characteristics According to Clinical GG1 and GG2

Among the 1,590 patients who underwent RP between 2009 and 2018 in our institution, 1,508 men with complete clinicopathologic records were recruited in this study. Of these, 322 (21.3%) were clinical GG1 cases and 170 (11.2%) were clinical GG2 cases. Table 1 summarized the baseline clinical characteristics of all clinical GG1 and clinical GG2 cases. The mean age of patients in the GG1 group was 67.2 ± 0.4 years old and 65.7 ± 0.8 years old in the GG2 group. GG2 cases exhibited higher level of PSA and PSAD compared with GG1 cases. A larger proportion of men in GG2 had higher clinical stage than those in GG1.

**TABLE 1:** Baseline patient characteristics.

	Clinical GG1 (n = 322)	Clinical GG2 (n = 170)	*p* Value
Age at diagnosis, mean ± SD (years)	67.2 ± 0.4	65.7 ± 0.8	0.188
BMI, mean ± SD (kg/m2)	23.8 ± 0.3	24.2 ± 0.1	0.391
Prostate volume on TRUS, mean ± SD (ml)	36.8 ± 0.9	34.1 ± 1.5	0.020
Baseline PSA, mean ± SD (ng/ml)	7.6 ± 1.8	11.0 ± 2.8	<0.001
PSA density, mean ± SD	0.24 ± 0.09	0.34 ± 0.12	<0.001
No. positive cores mean ± SD	2.5 ± 0.9	2.5 ± 0.8	0.905
Clinical T stage, *n* (%)			<0.001
T1c	48 (14.9)	6 (3.5)	
T2	245 (76.1)	138 (81.2)	
T3 and above	29 (9.0)	26 (15.3)	

BMI, body mass index; GG1, grade group 1 prostate cancer; GG2, grade group 2 prostate cancer; PSA, prostate specific antigen; SD, standard deviation; TRUS, transrectal ultrasound.

### Postoperative GS Upgrading and Upstaging in Clinical GG1 and GG2

Table 2 illustrated the upgrading of GS and proportion of EPE and SVI at RP in the group with clinical GG1 and clinical GG2 cases. Redistribution of GGs before and after surgery was presented in Table 3. In sum, 78 (24.2%) out of 322 men in clinical GG1 group demonstrated an upgrading of GS on their RP pathology. Two hundred forty-four men (75.8%) showed no GS change or downgrading. In the clinical GG2 group, 55 (32.4%) out of the 170 patients exhibited GS upgrading. 112 men (65.9%) had no GS change and 3 (1.7%) of them downgraded. Of the 322 men with clinical GG1 cancer, 37 (11.5%) exhibited EPE and none had SVI.

**TABLE 2:** Postoperative pathological changes in clinical GG1 and GG2.

	Clinical GG1 (n = 322)	Clinical GG2 (n = 170)
Total no. GS upgrading (%)	78 (24.2)	55 (32.4)
EPE, no. (%)	37 (11.5)	49 (28.8)
SVI, no. (%)	0 (0)	13 (7.6)
PSM, no. (%)	69 (21.4)	44 (25.9)

EPE, extraprostatic extension; GG1, grade group 1 prostate cancer; GG2, grade group 2 prostate cancer; GS, Gleason score; PSM, positive surgical margin; SVI, seminal vesicle invasion.

**TABLE 3:** Redistribution of grade groups before and after surgery.

	≤cG5^a^	cGG1^b^	cGG2	cGG3	cGG4	cGG5
≤ pG5^a^	0	21	0	0	0	0
pGG1^b^	5	223	3	11	5	1
pGG2	0	53	112	18	6	0
pGG3	0	19	44	N.A.	N.A.	N.A.
pGG4	0	6	11	N.A.	N.A.	N.A.
pGG5	0	0	0	N.A.	N.A.	N.A.

^a^ G5 indicates Gleason score 3 + 2 = 5 or 2 + 3 = 5 prostate cancer.

^b^ GG1 indicates Gleason score 3 + 3 = 6 prostate cancer.

cGG, clinical grade group; pGG, pathological grade group; NA, not assessed.

### Distribution of Adverse Pathological Features in Pathological GG1 and GG2

Among the 1,508 men recruited in our study, 248 (16.4%) had pathologic GG1 and 189 (12.5%) had pathologic GG2. The analysis of pathologic characteristics in pathological GG1 and GG2 cancers was outlined in Table 4. As shown in Table 3, pathologic GG1 cases (n = 248) included upgraded cases (n = 5) and downgraded cases (n = 20, from clinical GG2, GG3, GG4 and GG5), in addition to 223 men with clinical GG1. Pathologic GG2 cases (n = 189) included 53 upgraded cases (from clinical GG1), 24 downgraded cases (18 with clinical GG3 and 6 with clinical GG4), in addition to 112 cases with clinical GG2. The incidence of EPE and SVI in pathological GG1 was 8.9% and 0%, respectively. The incidence of LVI and PNI in the pathological GG1 cases was 28.6% and 2.8%, respectively. There were 115 cases (60.8%) in pathological GG1 that demonstrated tumor multiplicity. When we compared the postoperative findings between pathological GG1 and GG2, pathological GG2 men had statistically higher incidence of LVI and PNI compared to GG1 men.

**TABLE 4:** Pathologic characteristics in pathological GG1 and GG2.

	Pathological GG1 (n = 248)	Pathological GG2 (n = 189)	*p* Value
PSA, mean ± SD (ng/ml)	7.1 ± 1.8	8.5 ± 0.8	0.040
PSA density, mean ± SD	0.21 ± 0.09	0.28 ± 0.12	<0.001
EPE, *n* (%)			
Yes	22 (8.9)	42 (22.2)	<0.001
No	226 (91.1)	147 (77.8)	
SVI, *n* (%)			
Yes	0 (0)	10 (5.3)	<0.001
No	248 (100)	179 (94.7)	
Multiplicity, *n* (%)			
Yes	151 (60.8)	139 (73.5)	0.007
No	97 (39.2)	50 (26.5)	
Tumor volume, mean ± SD (ml)	1.9 ± 0.6	4.2 ± 1.6	<0.001
LVI, *n* (%)			
Yes	7 (2.8)	18 (9.5)	0.005
No	241 (97.2)	171 (90.5)	
PNI, *n* (%)			
Yes	71 (28.6)	119 (62.9)	<0.001
No	177 (71.4)	70 (37.1)	
PSM, no. (%)	45 (18.1)	28 (14.8)	0.426

EPE, extraprostatic extension; GG1, grade group 1 prostate cancer; GG2, grade group 2 prostate cancer; LVI, lymphovascular invasion; PNI, perineural invasion; PSA, prostate specific antigen; PSM, positive surgical margin; SD, standard deviation; SVI, seminal vesicle invasion.

Further analysis comparing the pathological features between pathological GG1 T3 and GG2 T2 was presented in Table 5. There were no statistical differences in the incidence of LVI and PNI between the two groups.

**TABLE 5:** Pathologic characteristics in pathological GG1 and T3 vs. GG2 and T2.

	Pathological GG1 and T3 (n = 22)	Pathological GG2 and T2 (n = 147)	*p* Value
LVI, *n* (%)			
Yes	1 (4.6)	10 (6.8)	1.000
No	21 (95.4)	137 (93.2)	
PNI, *n* (%)			
Yes	10 (45.4)	70 (47.6)	1.000
No	12 (54.6)	77 (52.4)	
Multiplicity, *n* (%)			
Yes	15 (68.2)	106 (72.1)	0.898
No	7 (31.8)	41 (27.9)	
Tumor volume, mean ± SD (ml)	3.8 ± 4.3	3.3 ± 2.4	0.320

GG1: grade group 1 prostate cancer, GG2: grade group 2 prostate cancer, LVI: lymphovascular invasion, PNI: perineural invasion, SD: standard deviation.

**FIGURE 1:**
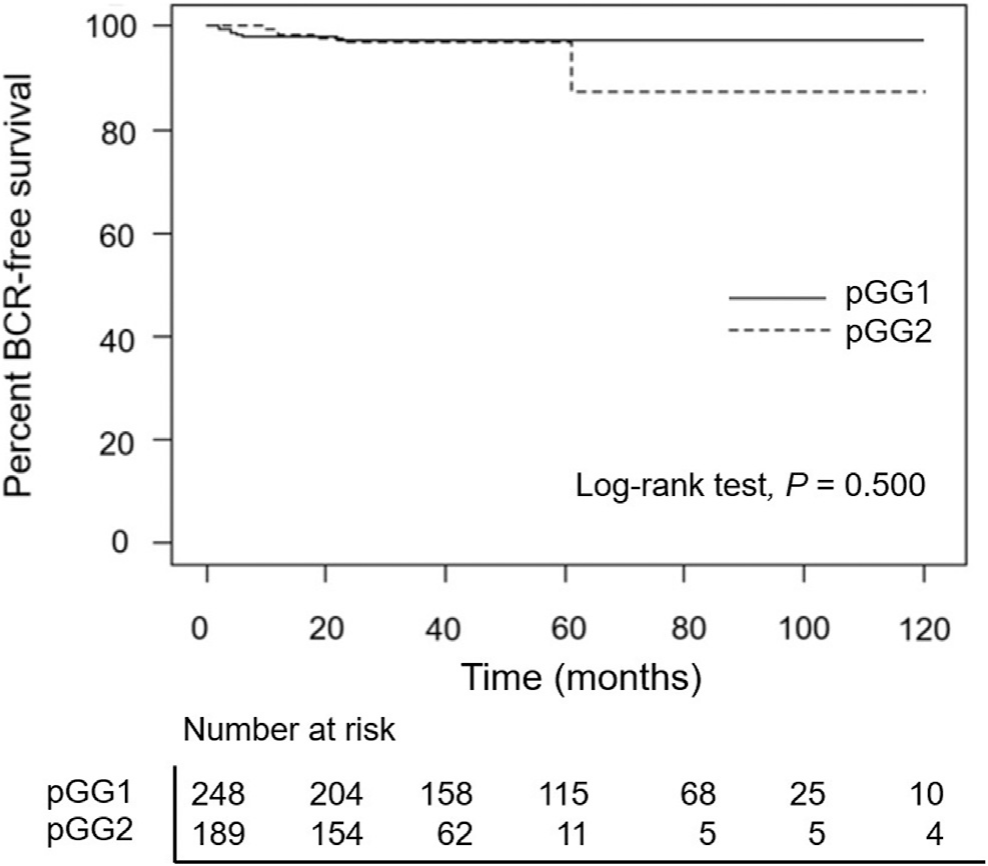
Kaplan–Meier curves of biochemical recurrence-free survival according to pathological groupings. pGG1: pathological grade group 1 prostate cancer, pGG2: pathological grade group 2 prostate cancer.

A total of 6 men (2.4%) in pathological GG1 and 6 men (3.2%) in pathological GG2 cases experienced BCR. BCR-free survival was observed in 97.4% (at 5- and 10-years) of patients in the pathological GG1 group and 97.0% and 87.3% (at 5- and 10-years, respectively) in the pathological GG2 group (Figure 1). BCR-free survival rates were similar (*p* = 0.500) between the two groups. When we analyzed the pathological GG1 cases according to T stage, BCR was observed in 4 men in pathological GG1 T2 (n = 226) and 2 men in GG1 T3 (n = 22) group.

## Discussion

The main findings of this retrospective study were as follows: 1) A considerable number of patients diagnosed with GG1 from their biopsy experienced postoperative GS upgrading and upstaging. 2) In pathological GG1 cases, there have also been quite a few adverse pathological features, including locally advancing, LVI, and PNI. 3) Especially at pT3 stage, pathological GG1 cases showed BCR frequently. 4) Taken together, it seems that GG1 still possess its respectable position as a group of cancer with aggressiveness.

As for the incidence of postoperative GS upgrading, it has been notable that Gleason score 3 + 3 = 6 tumors on prostate biopsy are upgraded at RP at a rate of approximately 20%–35% [14, 15]. However, most of these results were from studies conducted in the time of pre-ISUP 2014. At the time of post-ISUP 2014 grading system, current GG1 (so-called pure Gleason score 3 + 3 = 6) tumors have a lesser aggressive nature compared to that in pre-ISUP 2014 era because tumors with all cribriform patterns are now assigned to Gleason score 4. Therefore, analysis of GS upgrading of GG1, not Gleason score 3 + 3 = 6 in pre-ISUP2014 era, is important. In our study, the incidence of GS upgrading in clinical GG1 cases was 24.2%. Additionally, even Anderson et al. [11] who was in favor of removing cancer label from GG1, expressed concerns on GS upgrading at RP in clinical GG1 cases. It is noteworthy that the postoperative GSs from clinical GG1 in our study were not only Gleason score 3 + 4 = 7. As illustrated in Table 3, a considerable proportion of patients revealed a final pathologic Gleason score 4 + 3 = 7 tumor (24%, 19 out of 78 total upgraded cases) and there were also final pathologies ≥ Gleason score 4 + 4 = 8 (7.7%, 6 out of 78 upgraded cases).

Another important finding is the incidences of EPE in clinical or pathological GG1 cases. In our study EPE was seen in 11.5% of clinical GG1 and 8.9% of pathological GG1 groups. In 2017 Anderson et al. [11] reported extremely rare incidence (0.28%) of EPE and SVI (0%) in pathological GG1 cases. More recently however, Hassan et al. [15] reported their results in favor of continuing to use the term cancer for this lesion. Analysis of their large RP cohort revealed that the incidence of EPE is not rare (Total EPE incidence was 6.3%, focal EPE 3.9% plus no-focal EPE 2.4%) in the pathological GG1 cases. The authors indicated the possible selection bias (e.g. exclusion of cases enriched for potential GS6 with EPE) of the previous report [11]. In our study, 8.9% of all pathological GG1 cases exhibited EPE, which seemed to be of higher incidence when compared after reviewing recent reports [11, 15]. As regards GG2 cases, EPE was observed in about 20% of the cases in our study.

LVI has been reported to be a predictor of disease progression in both pT2 and pT3 prostate cancers; however, several conflicting reports exist [16–18]. Mitsuzuka et al. [16] reported its good correlation with BCR in pT2N0 negative surgical margin as well as all patients. Based on Korean data, Park et al. [17] analyzed 1,622 men from the K-CaP database and reported that LVI is one of the most powerful adverse prognostic factors for BCR in men with pT3 N0. Other Korean researchers have reported the association of LVI with lymph node metastasis [19]. Herman et al. [20] suggest the inclusion of LVI in nomograms to predict disease recurrence in prostate cancer, supporting the recommendation from the *Cancer* Committee of the College of American Pathologists. Although the incidence was relatively low, LVI was present (2.8%, 7 out of 248) in pathological GG1 men in our analysis. These include five men with pT2 and two with pT3 disease.

Similarly, PNI seemed to play a role as a predictor of BCR in men with prostate cancer. In 2018, Zhang et al. [21] performed a systematic review and meta-analysis and concluded that PNI is associated with higher risk of BCR. From the Korean data, Kang et al. [22] studied 2,394 men who underwent RP and reported PNI as an independent predictor for BCR. However, there were also contradicting reports [23]. In the present study, 28.6% of our pathological GG1 cases had PNI.

Taken together, considering our findings from the analysis using both 1) GG1 from biopsy and 2) pathological GG1 among Korean population, it seems that the aggressiveness of GG1 tumor is not so neglectable as currently known. We have concerns on the current belief that GG1 is almost always clinically insignificant, and we think such misconception would result in missing of opportunity for cure of significant cancer.

The distinctive feature of our study is that, to the best of our knowledge, this is the first report on this issue in the Korean population. Few data are available which analyzed the clinicopathological features of contemporary GG1 cases among Asian population. More importantly, GS upgrading in clinical GG1 was not assessed in previous literatures [11, 13]. The current study not only provides adequate preliminary information to literatures about contemporary guidelines (including AS protocols for low-risk disease) among Asian populations but also support the idea in favor of continuing to use the term “cancer” for GG1 lesion.

It is well known that patients with prostate cancer in Asian countries present with more aggressive pathologic features, compared to Western men [24, 25]. Especially for Korean patients, prostate cancer in Korean men is known to be more aggressive and exhibit poorer differentiation, regardless of the initial serum PSA level or clinical stage at presentation, than that of Western men [26]. In our study, incidences of EPE in pathological GG1 cases seemed to be slightly higher than that of Western studies [11, 15] despite the lack of statistical power. These findings might suggest the need for future well-designed study with a larger number of Asian patients.

In addition, we performed further analysis to compare the pathological characteristics between pathological GG1 T3 vs. GG2 T2 (Table 5.) This analysis was done in order to identify the status of pathological GG1 which has T3 stage. Our result showed no statistical differences in the incidence of LVI and PNI between the two groups, which means that the status of GG1 T3 cancer is roughly equivalent to GG2 T2. We think this finding would be another point against several authors who suggested the removal of the cancer label from this lesion simply because it is GG1. For reference, when we analyzed the BCR-free survival in pathological GG1 T3 cases (n = 22), 2 cases (9.1%) showed BCR. This also is a finding which supports that GG1 possess its respectable position as a group of cancer, considering that the incidence of EPE was not rare in pathological GG1 cases and BCR occurred frequently especially when they had pT3 stage.

**FIGURE 2:**
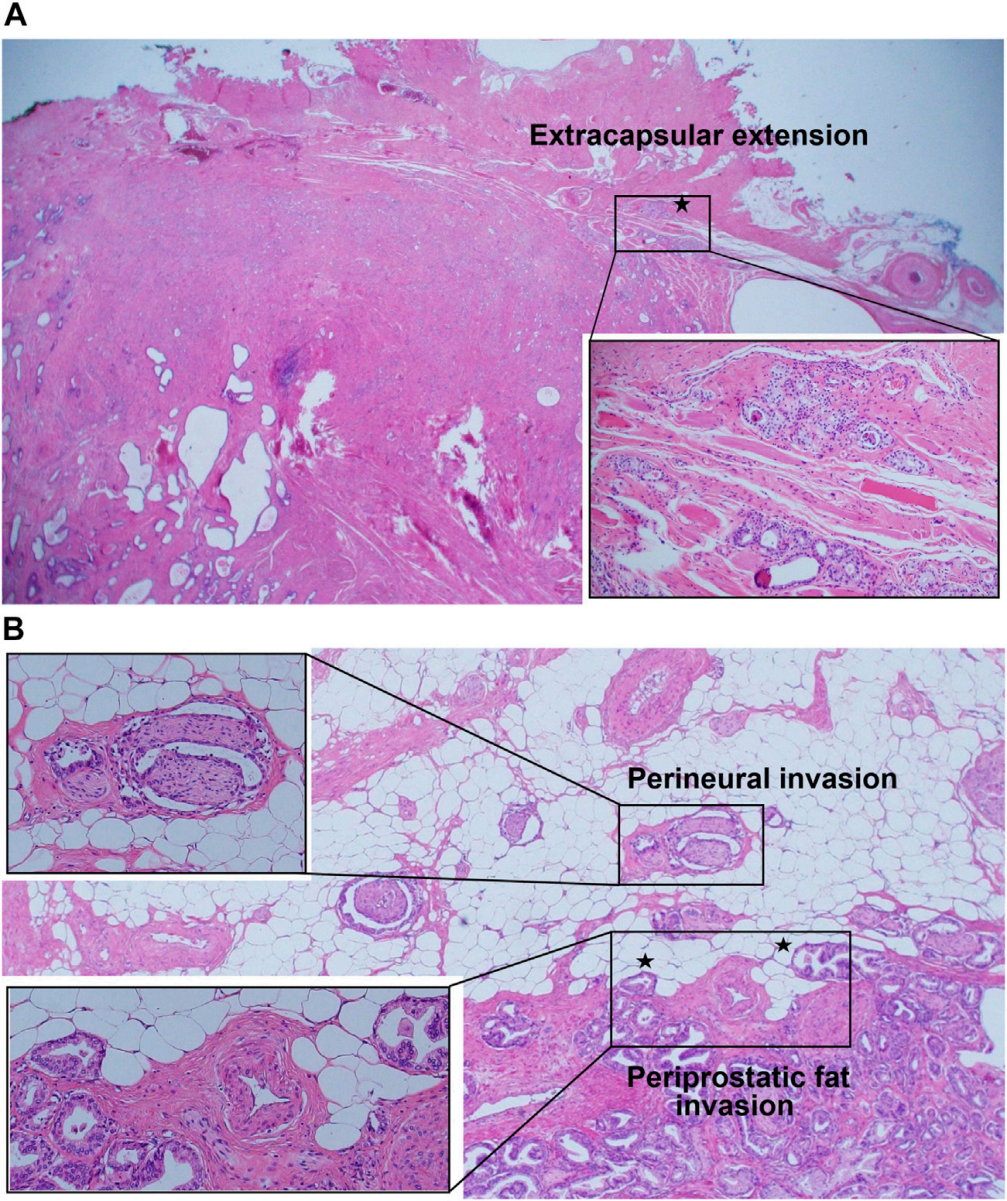
**(A)** Extension of grade group 1 tumor beyond the fibrous capsule of prostate (extracapsular extension). **(B-1)** Grade group 1 tumor surrounding nerves in the neurovascular bundle beyond the boundary of normal prostatic glandular tissue (perineural invasion). **(B-2)** Presence of nodular extensions of grade group 1 tumor bulging beyond the periphery of prostatic stroma (at the outer edge), abutting or within periprostatic adipose tissue (periprostatic fat invasion).

Several key aspects regarding the aggressiveness of GG1 cancer are as follows: 1) There has been several reports demonstrating that GG1 tumors shares histological [15, 27, 28] and molecular [27, 28] features with higher grade prostate cancers. Histologically GG1 shows the loss of basal cells and invasion into the stroma, which is seen in higher grade tumors. In addition, some authors highlighted the ability of GG1 cancers to invade tissues beyond the prostate and infiltrate around nerves or periprostatic fat. Kulac et al. [28] suggested that such findings would be common features between GG1 and higher grade cancers, and clear sign of invasive potential. Similarly, Hassan et al. [15] emphasized that EPE could be a histological feature typically related to aggressive behavior of prostate cancer. This is in line with our study demonstrating EPE, PNI and periprostatic fat invasion of GG1 tumors (Figure 2). Also, many molecular alterations including TMPRSS2-ERG fusions, chromoplexy and PTEN alterations were found in GG1 cancers, as well as higher grade cancers [27, 28]. These findings suggest common molecular pathways and mechanisms between GG1 tumor and higher grade tumors, for the development of many somatic genomic changes. Considering the histological and molecular backgrounds, it would be difficult to guarantee the safety of GG1 cases unless they are carefully monitored (e.g. sequential biopsies during years) or properly treated, because there is a potential for progression of the disease. 2) Another important aspect is the presence of GS upgrading at RP specimen, even up to Gleason score 4 + 4 = 8. In the absence of RP we cannot know with certainty whether the patient has only GG1 cancer. Especially when a physician has the misconception that GG1 is almost always insignificant, it is possible that the physician underestimates the disease severity for clinical GG1 cases. This carries the risk of undertreatment of a significant disease.

Limitations of current study include the small number of cases and its retrospective design. Another limitation could be that a single pathologist evaluated the slides of pre-ISUP 2014 era, which was related to a potential bias due to lack of interobserver agreement. Also, we did not subdivide the EPE into focal and non-focal EPE although this does not diminish our conclusion. For reference, results from Hassan et al. [15] consisted of focal and non-focal EPE incidence in each study group. Several reports demonstrated good correlations of focal or non-focal EPE with BCR, although its definition was subjective in their study.

Our study is also limited in that there was no significant difference in BCR-free survival between pathological GG1 and GG2 group, despite higher incidence of adverse pathologic features with pathological GG2 group than with GG1 group. It is possible such finding was due to the small number of patients who had adequate person year in pathological GG2 group (median follow-up periods of 32 months in our pathological GG2 group), and we need more time for data maturation to get more reliable survival difference. However, this does not diminish our conclusion which demonstrates the aggressiveness of GG1 tumor.

In conclusion, this current study represents a retrospective analysis of clinicopathological features of contemporary GG1 prostate cancer in Korean populations. Based on our findings, it seems that GG1 still possess its respectable position as a group of cancer with aggressiveness. These findings should be kept in mind when deciding for treatment options for prostate cancer patients in the Asian populations.

## Data Availability

The raw data supporting the conclusions of this article will be made available by the authors, without undue reservation.
